# Maturation-based Corrective Adjustment Procedures (Mat-CAPs) in youth swimming: Evidence for restricted age-group application in females

**DOI:** 10.1371/journal.pone.0275797

**Published:** 2022-10-07

**Authors:** Clorinda Hogan, Shaun Abbott, Mark Halaki, Marcela Torres Castiglioni, Goshi Yamauchi, Lachlan Mitchell, James Salter, Michael Romann, Stephen Cobley

**Affiliations:** 1 Sydney School of Health Sciences, Faculty of Medicine & Health, The University of Sydney, Camperdown, New South Wales, Australia; 2 Queensland Academy of Sport, Nathan, Queensland, Australia; 3 Swimming Australia Limited, Sunnybank, Queensland, Australia; 4 Swiss Federal Institute of Sport Magglingen, Magglingen, Switzerland; Universidade Federal do Rio Grande do Sul, BRAZIL

## Abstract

Inter-individual differences in maturation-associated development can lead to variations in physical performance, resulting in performance (dis)advantages and maturation selection bias within youth sport systems. To address such bias and account for maturational differences, Maturation-based Corrective Adjustment Procedures (Mat-CAPs) could be beneficial. The present study aimed to: (1) determine maturity timing distributions in youth female swimming; (2) quantify the relationship between maturation status and 100-m front-crawl (FC) performance; (3) implement Mat-CAPs to remove maturational influences upon swimming performance. For Aim 1 and 2, participants were 663 female (10–15 years) swimmers who participated in 100-m FC events at Australian regional, state, and national-level competitions between 2016–2020 and underwent anthropometric assessment (mass, height and sitting height) to estimate maturity timing and offset. For Aim 3, participants aged 10–13 years were categorised into maturity timing categories. Maturity timing distributions for Raw (‘All’, ‘Top 50%’ and ‘Top 25%’) and Correctively Adjusted swim times were examined. Chi-square, Cramer’s *V* and Odds Ratios determined the presence of maturation biases, while Mat-CAPs identified whether such biases were removed in targeted age and selection-groups. Results identified that between 10–13 years, a significantly higher frequency of ‘early’ maturers was apparent, although tapered toward higher frequencies of ‘Late-normative’ maturers by 14–15 years. A curvilinear relationship between maturity-offset and swim performance was identified (*R*^*2*^ *=* 0.51, p<0.001) and utilised for Mat-CAPs. Following Mat-CAPs application, maturity timing biases evident in affected age-groups (10–13 years), and which were magnified at higher selection levels (‘Top 50%’ & ‘25%’ of swim performances) were predominantly removed. Findings highlight how maturation advantages in females occurred until approximately 13 years old, warranting restricted Mat-CAPs application. Mat-CAPS has the potential to improve female swimmer participation experiences and evaluation.

## Introduction

In the last 10–15 years, the process of systematically identifying and developing precocious young athletes has become increasingly professionalised [1]. However, ‘practice in the field’ often fails to consider the consistent research informed proposition that athlete development is a multifactorial, nonlinear, process which occurs over time [2–5]. For instance, technical (e.g., motor coordination), physical (e.g., aerobic/anaerobic capacities), social (e.g., coaching expertise) and environmental (e.g., quality and structure of training) factors—and their dynamic interactions over time—have to be considered within and across the sport systems [6–10]. On this basis, any processes or factors which undermine or contra-indicate the capability to accurately identify genuinely skilled athletes need to be addressed. The biological process of maturational variation is one inter-individual factor known to affect participation, talented identification, and performance development [11–14].

Within age-grouping organisational processes in youth sport, chronological age variation unintentionally introduces participation inequalities, performance (dis)advantages and selection biases [15]. But inter-individual differences are further magnified by maturity-associated anthropometric and physiological variation. While growth is continuous in males and females until reaching adult maturity at approximately 18–20 and 16–18 years of age, respectively [16], maturation during adolescence is associated with time-periods of re-accelerated growth, where timing and tempo (growth rate) varying between individuals. For males, Peak Height Velocity (PHV; i.e., maturation high-point) occurs ‘normatively’ around 13.5–14.0 years [17], with a PHV gain of 6–10.5 cm/year estimated around 11.5–12 years [16–18], but may occur between 12 (i.e., earlier-maturing) and 15 (i.e., later-maturing) years of age. The ‘Normative’ female will gain an estimated 6–10.5 cm/year between 11.5–12 years, however, the timepoint of PHV can vary from 11 (i.e., earlier-maturing) to 14 (i.e., later-maturing) years of age [16–18]. Alongside anthropometric change, physiological changes are apparent. Males experience greater increases in, muscular strength and lean muscle mass [19, 20]. On the other hand, while females also accrue strength and muscle mass (although to a lesser extent), they also accrue greater fat mass [21], and may develop greater anthropometric variability (e.g., hip growth, torso shape and breast development) which may counteract against athletic performance [22, 23]. For instance, studies have shown how higher percentages of accumulated fat mass were associated with decreased performance in females [22–24], while another study has highlighted how increased chest depth at ages post-PHV was associated with decreased performance [11]. Prior youth swimming studies identify how anthropometric factors alone may explain up to approximately 20% of swimming performance variation in youth swimmers with reference to specific stroke events and developmental stages [8, 25, 26]. Similarly, energetic indices have been shown to explain approximately 15% of performance variation [8, 26]; while biomechanical and technique factors explain up to 85% across an annual training season. Together, the changing development (and interactions) of these indices may help account for the occurrence of superior (or plateauing) swim performance within and across youth age-group stages [27]. For instance, independent studies illustrate how greater height and lean body mass are predictive of aerobic power, muscular strength, endurance and speed [19]. In swimming, power and strength aid leg-kick force, stroke efficiency [28] and upper body propulsive power [29]. As a result, swimmers with advanced maturity-related anthropometric and physiological characteristics, are likely afforded performance advantages [30]. However, such one-directional development may not occur in female swimmers during or post-maturation, and the particular effects of maturation upon female anthropometric and physiological characteristics need to be carefully considered.

Based on the predicted benefit of advanced maturation on male swimming, Abbott & colleagues [30] previously identified significant overrepresentations of ‘Early’ maturing swimmers in youth competition. Notably, there was a complete absence of ‘Late’ swimmers. With the intent to mitigate against the influence of maturity, Abbott et al., then uniquely developed Maturation-based Corrective Adjustment Procedures’ (Mat-CAPs), adjusting raw performance times to control for maturation status differences in swim performance. Following performance time adjustment, maturity distribution across age-groups and selection levels were re-examined, identifying no maturity biases. As such, Mat-CAPs was able to remove the influence of maturation on performance differences.

Given the succes of initial Mat-CAPs application in males, follow-up questions were immediately raised. For instance, in what developmental age-groups and stroke events should (and should not) Mat-CAPs be applied? As females expectedly transition through maturation chronologically earlier with potential subsequent differential impacts swim performance, when would Mat-CAPs be applicable? Without pre-existing data available on females, the rationale to determine when (i.e., age-groups) maturity-related participation inequalities and performance (dis-)advantages was identified. Further, with such verification, determination of when Mat-CAPs would (not) be applicable could also be identified. Thus, study aims were to: *Aim 1*—examine maturity timing distributions in a large sample of female swimmers; *Aim 2*—quantify the stroke and distance-specific relationship between maturation status and swim performance across age-groups based on official competitive data; *Aim 3*—apply Mat-CAPs—where age-appropriate—to determine when maturation-related biases in the female 100-m FC swimming could be removed [27, 31].

## Materials and methods

### Participants

Following ethics approval (App No: 2018/762) from The University of Sydney Human Research Ethics Committee, as well as parental and swimmer informed written consent, participants were competitive female swimmers (*N* = 663) competing within the 10–15 years. Participant characteristics are summarised in Table 1. Swimmers competed in official long-course 100-m FC events at age-group Australian regional (*N* = 9), state (*N* = 7) or national (*N* = 1) competitions. All 100-m FC performance was extracted from Australian long course competitions between 2016–2020 (inclusive).

**Table 1:** Youth female participant (*N* = 663) characteristics and 100-m FC mean performance time.

Variable	M	SD	Minimum	Maximum
Age (years)	13.4	1.40	10.1	15.98
Body mass (kgs)	53.4	9.79	26.1	94.6
Height (cm)	163.3	8.49	133.5	183.6
Sitting height (cm)	85.9	4.87	71.1	103.6
Leg length (cm)	77.4	5.04	60.8	95.3
APHV Mirwald (years)	11.9	0.54	10.5	13.6
APHV Moore (years)	11.8	0.41	10.9	13.1
PPAS (%)	96.0	3.64	82.2	100.0
100-m FC (sec)	67.3	5.49	56.0	86.4

***Table Notes*:** M **=** Mean; SD = Standard Deviation, kgs = Kilograms; cm = Centimetres; APHV = Age at Peak Height Velocity; Mirwald = APHV estimated via the equation developed by Mirwald and colleagues [21]; Moore = APHV estimated via the equation developed by Moore and colleagues [34]; FC = Front-crawl; PPAS = Predicted Adult Stature; sec = Second.

#### Aim 1: Maturity inequalities in age-group competition participation

*Methods*. At respective swimming events, swimmers reported demographic details and underwent anthropometric measures of body mass, height and seated height. Trained researchers applied measurement procedures adherent with the International Society for the Advancement of Kinanthropometry standards [32]. Body mass (kg) was assessed using digital scales (A&D UC-321, Tokyo, Japan) to the nearest 0.1 kg. Standing height (cm) and sitting height (cm) were taken using a portable stadiometer (SECA 217, Hamburg, Germany), applying the stretch stature method [32]. All measures were taken in duplicate with mean values recorded. A third measure was taken if measures differed by 0.4 cm and 0.4 kg, respectively.

Somatic maturation was estimated using sex-specific somatic equations to estimate Years from Peak Height Velocity (YPHV) provided by Mirwald et al. [33], Moore et al. [34] and Percentage of Predicted Adult Stature (PPAS) provided by Sherar et al. [35] However, as the sample contained multiple females who’d attained full adult height (i.e., PPAS = 100%), but who were still developing physically (e.g., body mass; strength gains etc.), PPAS estimates were not utilised as an indicator of maturity status. The alternatives were considered, given Aim 3, and the need to differentiate maturation status variability pre- and post-PHV (including those at full adult height). Previously, the Moore et al. [34] method has been criticised for reducing true variation above and below estimates of PHV [36, 37] which was also seen within this sample (see Table 1), therefore, the Mirwald et al. [33] equation was applied. The Mirwald et al. [33] equation also has known error associated with over-fitting. Still, it utilises more variables to provide a more accurate estimation of ‘Early’ and ‘Late’ maturing females relative to the Moore et al. [34] equation [37]. Thus, the Mirwald et al. [33] equation was utilised to estimate Years from Peak Height Velocity (YPHV), specifically:

$$\begin{array}{l} YPHV=-9.376+0.001882\times (leg\;length\times sitting\;height)+0.0022\times (age\;\times \;leg\;length)+0.005481\times \\ (age\;\times \;sitting\;height)-0.002658\;\times \;(age\;\times \;body\;mass)+0.07693\times \left(\frac{body\;mass}{height}\times 100\right) \end{array}$$

Age at PHV (APHV) was calculated by subtracting YPHV from decimal age on the date of measurement to estimate maturity timing. For example, a female with a decimal age of 11.50 years and an estimated YPHV of -0.75 years, had an estimated APHV of 12.25 years. On this basis, swimmers were then categorised into maturational-timing categories based on normative population female APHV values (11.90±1): ‘Early’ (≤10.90 APHV), ‘Normative’ (10.91–12.89 APHV) and ‘Late’ (≥12.90 APHV) [16, 38].

**Fig 1:**
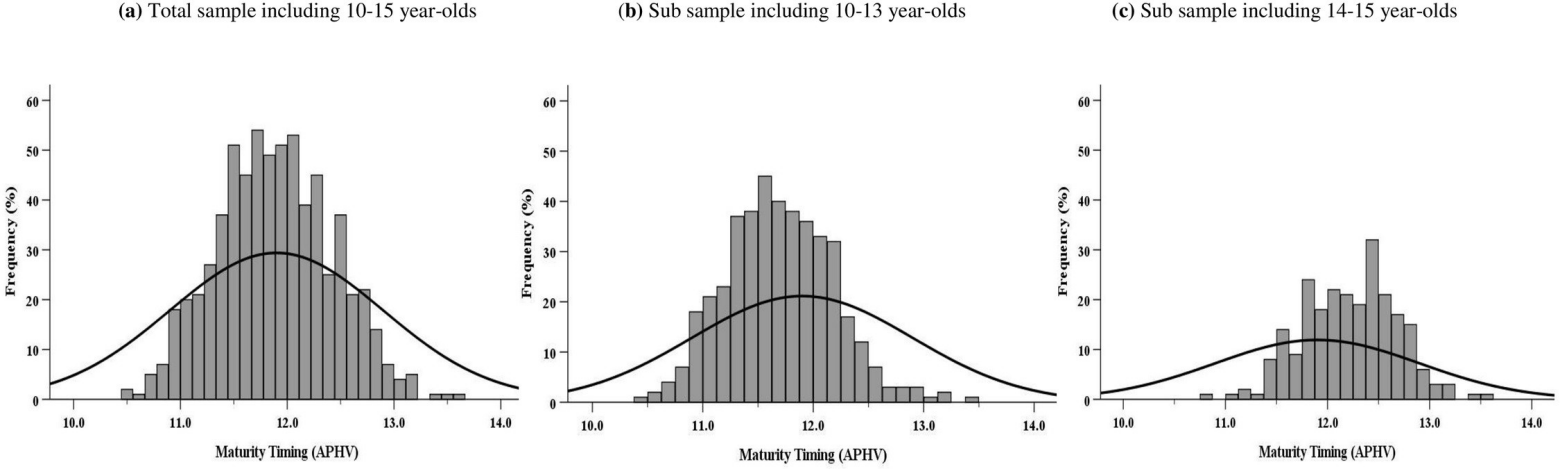
Frequency distributions of maturity timing (APHV) for *N* = 663 female 100-m FC swimmers plotted relative to expected female normative population APHV distributions. Fig 1a illustrates ‘All’ sample distribution; 1b illustrates the distribution of 10–13 year-olds; and 1c illustrates the distribution of 14–15 year-olds.

*Data analysis*. Expected percentage distributions in the ‘Normative’ maturational-timing category was 68.2%, with 15.85% expected in the ‘Early’ and ‘Late’ categories. For study purposes, we sub-divided the ‘Normative’ category into two: ‘Early-normative’ (≤11.90 years APHV) and ‘Late-normative’ (<12.90 years APHV), with both categories expected to have 34.15% (68.2%/2) of the distribution. To determine the presence of maturational timing bias, observed distributions were compared against expected normative population distributions [16, 38] (see Fig 1). Distributions were analysed across ‘All’ the sample and according to age-group (10/11-15 years), reflecting competition structures (see S1 Table). Chi-square tests (*X*^*2*^; p < 0.05) compared distributions within maturity timing categories. Effect size magnitude was determined using Post-hoc Cramer’s *V*. For *df* = 3. A ‘small effect’ was indicated by 0.06< *V* ≤0.17; ‘medium effect’ 0.17< *V* <0.29; and a ‘large effect’ *V* ≥0.29 [39]. Odds Ratios (ORs) with 95% Confidence Intervals (CI) identified where maturity timing category distributions differed. ORs were calculated using the frequency of swimmers in a maturity-timing category relative to the ‘Late’ category (i.e., reference group), considerate of expected distributions (i.e., ‘Early’ and ‘Late’ = 15.85%; ‘Early-’ and ‘Late-normative’ = 34.15%). All statistical analyses were performed using IBM SPSS software for Windows (SPSS version 25, Chicago, IL, USA).

#### Aim 2: Relationship between maturity status and swimming performance

*Methods*. Swimmer maturation status (YPHV via Mirwald et al. [33]) was combined with 100-m FC race time data. Swimmers who competed in an official long-course 100-m FC events within approximately two days (range = ±14 days) of measurement were included. If a swimmer registered multiple performance times, the fastest time was utilised. The 100-m FC was examined due to the event being one of the most popular, with the highest participation numbers across Australia’s age-group competitions.

*Data analysis*. Data was initially screened using box plots with outliers removed (*n* = 24). Outlier swim performances were identified and excluded if race times exceeded 6 seconds above the regional level qualification times for each age-group. To determine the relationship between maturation status and 100-m FC performance, YPHV was plotted against 100-m FC performance time (seconds). Scatterplot visual inspection between YPHV and performance revealed a non-linear relationship; therefore, stepwise multiple regression was performed with YPHV and YPHV-squared as independent variables and performance time as the dependent variable. Pearson’s correlation coefficient (*r*) and adjusted coefficients of determination (*R*^*2*^) were calculated. The expected maturity status—performance relationship for females aged 10–15 years of age was obtained through a quadratic function (y = ax^2^ + bx + c) from unstandardised coefficients. The regression equation was subsequently used for Mat-CAPs in *Aim 3*.

#### Aim 3: Maturation status distributions according to selection level following Mat-CAPs

*Methods*. The unstandardised coefficients of the quadratic model generated from *Aim 2* were utilised to determine whether maturity-related performance influences could be removed. However, as Mat-CAPs requires a degree of swimmer distribution across maturity timing categories and given the clustering of maturity timing about the ‘Normative’ categories, swimmers were re-categorised according to sample M ± SD (11.89 ± 0.54). Initially, the observed frequency within maturity timing categories at each age-group (10/11-15 years) was examined (see Table 2). However, based on *Aim 1* results where maturation status biases were identified in the 10/11-12 years, with potentially uneven distributions at 13 years, Mat-CAPs application was only justified for these age-groups. As a reversal was evident from 14–15 years with overrepresentation of ‘Later’ maturing swimmers, Mat-CAPs application was not deemed necessary. A summary of maturity timing distributions for ‘All’ swimmers aged 10/11-13 years is summarised in Table 2. Age-group analyses were also conducted according to the ‘Top 50%’ and ‘Top 25%’ of overall performance times.

**Table 2:** Revised maturity timing distributions, chi-square, and odds ratio analyses of 424 female swimmers (10/11-13 years) according to raw ‘All’, ‘Top 50%’ and ‘Top 25%’, and correctively adjusted ‘Top 50%’ and ‘Top 25%’ of swim times.

Raw & Corrected Population	Age-Group	Total	Late	Late-Norm.	Early-Norm.	Early	*X* ^*2*^	*P*	*V*	ES cat.	Early v Late	Early-Norm v Late	Late-Norm v Late
											OR (LCI-HCI)	OR (LCI-HCI)	OR (LCI-HCI)
Raw All	10/11 years	124	9	19	57	39	**42.76**	**0.0001**	**0.34**	**Large**	**4.33 (1.66–11.29)**	**2.93 (1.22–7.11)**	0.97 (0.38–2.55)
	12 years	153	5	41	61	46	**38.67**	**0.0001**	**0.29**	**Large**	**9.20 (3.12–27.12)**	**5.66 (2.02–15.88)**	**3.80 (1.34–10.83)**
	13 years	147	12	62	54	19	**9.38**	**0.025**	**0.15**	**Small**	1.58 (0.63–3.99)	2.08 (0.94–4.63)	**2.39 (1.09–5.28)**
	14 years	144	35	59	46	4	**24.21**	**0.0001**	**0.24**	**Medium**	**0.11 (0.03–0.37)** ^**#**^	0.60 (0.31–1.18)	0.78 (0.41–1.50)
	15 years	95	55	29	11	0†	**134.51**	**0.0001**	**0.69**	**Large**	**0.009 (0.00–0.16)** ^**#**^	**0.09 (0.04–0.23)** ^#^	**0.24 (0.11–0.52)** ^**#**^
Raw Top 50%	10/11 years	62	4	9	27	22	**27.13**	**0.0001**	**0.38**	**Large**	**5.50 (1.38–21.96)**	3.13 (0.86–11.43)	1.04 (0.26–4.23)
	12 years	77	2	21	29	25	**23.29**	**0.0001**	**0.32**	**Large**	**12.5 (2.41–64.72)**	**6.72 (1.38–32.86)**	4.87 (0.98–24.17)
	13 years	74	5	26	30	13	4.92	0.178	0.15	-	2.60 (0.70–9.65)	2.78 (0.86–9.00)	2.41 (0.74–7.87)
Raw Top 25%	10/11 years	31	2	3	16	10	**15.20**	**0.002**	**0.40**	**Large**	5.00 (0.70–35.74)	3.71 (0.60–22.87)	0.69 (0.09–5.60)
	12 years	39	0†	4	19	16	**29.76**	**0.0001**	**0.50**	**Large**	**32.00 (1.55–660.71)**	17.63 (0.91–342.3)	3.71 (0.17–81.67)
	13 years	37	1	11	17	8	6.54	0.088	0.24	-	8.00 (0.75–85.85)	7.89 (0.84–74.27)	5.10 (0.53–49.39)
Correctively Adjusted Top 50%	10/11 years	62	6	12	30	14	**10.93**	**0.012**	**0.24**	**Medium**	2.33 (0.63–8.58)	2.32 (0.73–7.39)	0.92 (0.27–3.20)
	12 years	77	5	26	29	17	6.43	0.093	0.17	-	3.40 (0.95–12.16)	2.69 (0.84–8.65)	2.41 (0.75–7.80)
	13 years	74	7	30	27	10	3.19	0.364	0.12	-	1.42 (0.41–5.04)	1.79 (0.61–5.28)	1.98 (0.68–5.83)
Correctively Adjusted Top 25%	10/11 years	31	5	7	14	5	2.32	0.508	0.16	-	1.00 (0.17–5.82)	1.29 (0.30–5.70)	0.64 (0.14–3.12)
	12 years	39	2	12	18	7	4.73	0.193	0.20	-	3.50 (0.51–24.04)	4.17 (0.73–23.91)	2.78 (0.47–16.43)
	13 years	37	0†	18	10	5	**8.47**	**0.037**	**0.29**	**Large**	10.00 (0.43–233.28)	9.28 (0.45–190.92)	16.70 (0.84–334.04)

***Tables Notes*:** Late = Number with a late APHV (APHV >12.4 years); Late-Norm. = Number with a Late-Normative APHV (APHV < 12.4 years); Early-Norm. = Number with an Early-Normative APHV (APHV < 11.9 year); Early = Number with an Early Age of Peak Height Velocity (APHV < 11.4 years); † = Observed cell values of 0 were input as 0.5 to enable comparison of maturity timing categories; *χ*^*2*^ = Chi-Square value; *P* = Probability value; *V* = Cramer’s *V* effect size; ES cat. = Effect Size category; OR = Odds Ratio; LCI-HCI = Low & High 95% Confidence Intervals for maturation category comparisons;

# = overrepresentation of Late maturity timing category; **bold** = Significant Chi-square (*p* < 0.05; with *P*, *V* and effect size category reported) and/or significant ORs (with LCI-HCI) in specific maturation status group comparisons.

To determine whether Mat-CAPs removed maturity-performance biases within specific age-groups and selection levels, individual raw performance times were adjusted using expected differences estimated from the quadratic regression equation generated in *Aim 2*. Each swimmers’ performance time was adjusted according to the expected quadratic trend, to the point where all swimmers’ YPHV was matched to the most mature swimmer (highest YPHV) within each age-group. Frequency distributions were then re-examined using Correctively Adjusted performance times in the 10/11-13 age-groups and according to the ‘Top 50%’ and ‘25%’ of swim times (see Table 2).

*Data analysis*. Like *Aim 1*, maturity timing category distributions were examined and compared to expected distributions for ‘All’ swimmers and according to age-group (10/11-13 years) and selection level. Then Correctively Adjusted ‘Top 50%’ and ‘25%’ of swim times in corresponding age-groups were examined to determine if maturational biases had been removed. *X*^*2*^, Cramer’s *V*, and ORs with 95%CI identified the presence and magnitude of deviations between maturity timing categories relative to the ‘Late’ category and considerate of expected normative distributions (i.e., ‘Early’ and ‘Late’ = 15.85%; ‘Early-’ and ‘Late-normative’ = 34.15%).

## Results

### Aim 1: Maturity inequalities in age-group competition participation

Fig 1 illustrates the APHV frequency distribution for the swimmer sample (11.89 ± 0.54) and according to particular age-group categories. Within Fig 1A–1C, the solid black curve indicates the normal (expected) population APHV distribution (APHV = 12.00 ± 1.0). Across all participants, Fig 1A illustrates a tight clustering about norm values, with visible frequency reductions below expectation for ‘Early’ and ‘Late’ maturing tails of the distribution. Interestingly, when the sample was divided into 10–13 (Fig 1B) and 14–15 year-olds (1c), changes in distribution were evident. Fig 1B identified a higher frequency of ‘Early-normative’ maturers (11.70 ± 0.49). By contrast, for 14–15 year-olds, Fig 1C identifies an overrepresentation of ‘Late-normative’ females (12.22 ± 0.46).

Results from testing of maturational bias across ‘All’ swimmers and according to age-groups are summarised in S1 Table. Across all age groups, significant *χ*^*2*^ overrepresentations of ‘Early-normative’ and ‘Late-normative’ swimmers was evident with large effect sizes apparent (e.g., 12 years χ2 = 80.20, p<0.0001; ‘Early-normative’ v ‘Late’ OR = 46.48, 95%CI = 6.12–353.14; ‘Late-normative’ v ‘Late’ OR = 20.91, 95%CI = 2.72–160.76) relative to ‘Late’ (and ‘Early’) maturing swimmers (except 15 years age-group). Unexpectedly, there were no statistical differences between ‘Early’ and ‘Late’ maturing swimmers (‘Early’ v ‘Late’ OR = 1.00, 95%CI = 0.48–16.63). However, when examining age-groups, the frequency of ‘Earlier’ maturing swimmers decreased from 10/11 to 15 years, while the number of ‘Later’ maturing swimmers descriptively increased. Notably, by the 15 years age-group, an overrepresentation of ‘Late-normative’ maturing swimmers relative to other categories was apparent, emphasising a shift in maturity timing trends from 10–13 years to 14–15 years.

### Aim 2: Relationship between maturity status and swimming performance

**Fig 2:**
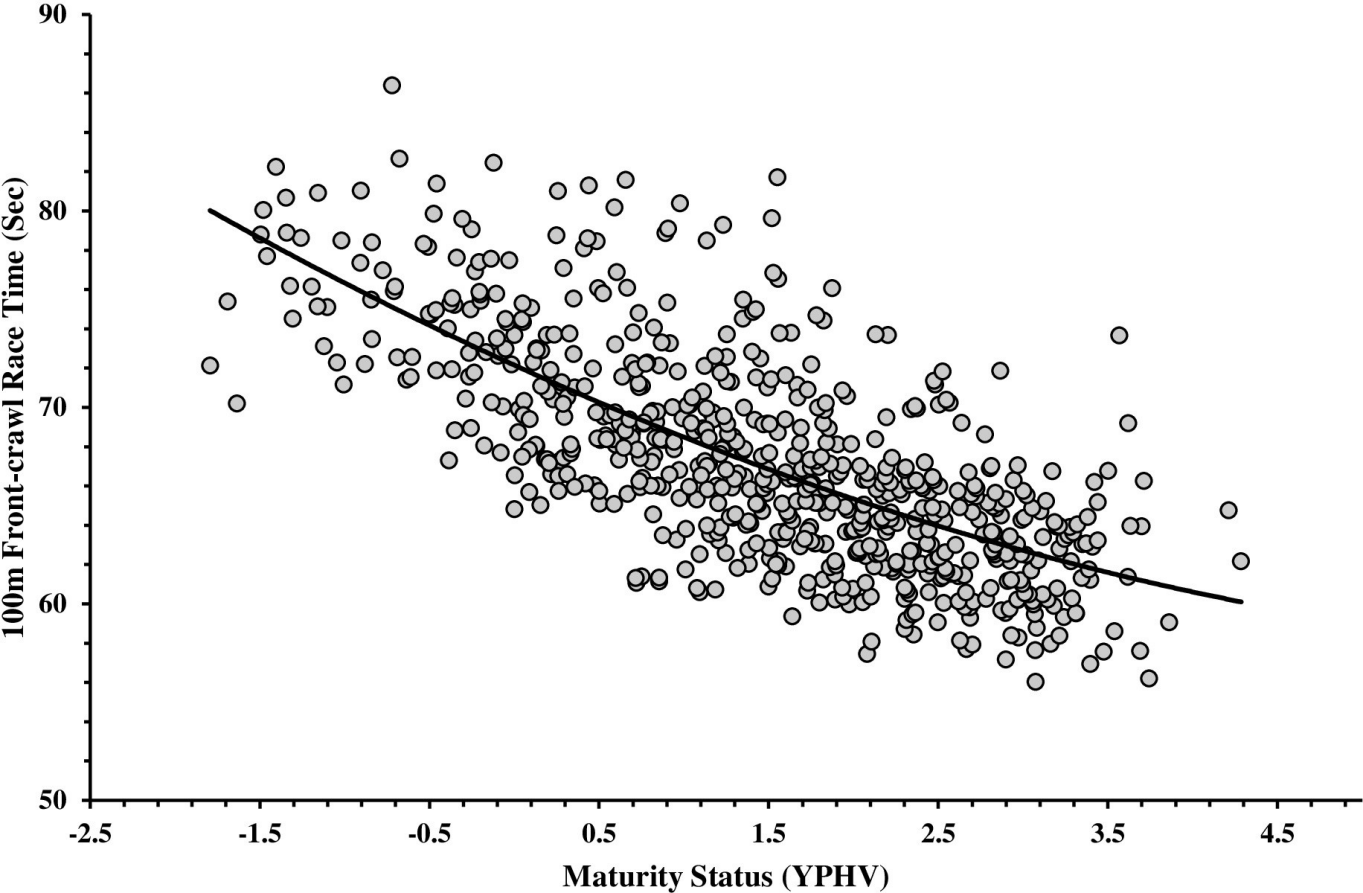
The curvilinear relationship between maturity status (YPHV) and 100-m FC performance time (sec) in females aged 10–15 years at regional-national level competitions.

The curvilinear (quadratic) relationship between maturation status (YPHV via Mirwald et al. [33]) and 100-m FC performance time across 10–15 years is summarised in Fig 2. YPHV (*F*(1,661) = 662.92, p < 0.001) and YPHV^2^ (*F*(2,660) = 339.57, p < 0.001), significantly predicted swim performance with *R*^*2*^ suggesting YPHV accounted for 50.70% (p = 0.003) of the variance in 100-m FC performance. Relationship estimates included: intercept = 72.15, *t* = 302.45, p < 0.001, *SE* = 0.24, 95%CI = 71.69–72.62; linear = -3.92, *t* = -15.08, p < 0.001, *SE* = 0.26, 95%CI = -4.43–-3.41; and, quadratic components = 0.26, *t* = 2.93, p = 0.003, *SE* = 0.09, 95%CI = 0.09–0.43.

### Aim 3: Maturation status distributions according to selection level following Mat-CAPs

Table 2 summarises results of the re-categorised sample-based maturity timing distributions for raw ‘All’ swimmers as well as according to age-group and selection level. Related to raw data, significant maturity timing bias towards the ‘Early’ and ‘Early-normative’ maturing was apparent, with large effect sizes in the 10/11-12 years age-groups. By 13 years, an overrepresentation of ‘Late-normative’ (v ‘Late’) swimmers emerged (OR = 2.39). At 14 and 15 years, more clear overrepresentations of ‘Late’ maturing swimmers relative to other maturity timing categories was evident. When reviewing raw performance selection level criteria, maturity timing bias toward the ‘Early’ maturing was again evident with large effect sizes at 10/11 and 12-years across both selection levels. For instance, in the ‘Top 50%’ at 12 years, the ‘Late-normative’ and ‘Late’ were underrepresented (‘Early’ v ‘Late’ OR = 12.5), while in the ‘Top 25%’ the effect size increased (‘Early’ v ‘Late’ OR = 32.00).

Table 2 also summarises results based on the Correctively Adjusted ‘Top 50%’ and ‘25%’ of swim times. Following Mat-CAPs application, results identify no significant maturation category bias away from expected maturity timing distribution for any of the targeted 10/11-13 age-groups. Only uneven distributions across all four maturity timing categories were identified (i.e., not specific to ‘Early’ or ‘Late’ categories within the sample). These findings suggest maturity biases identified based on raw performance assessment were now removed. See S1 Fig for a graphical illustration specific to the 12-year-old age-group.

## Discussion

When set against study *Aims 1–3*, findings firstly identified significant over-representations of ‘Early-normative’ and ‘Late-normative’ maturing female swimmers (aged 10–15) relative to expected distributions. In contrast with swim stroke and age-matched male samples, where overrepresentations of ‘Early’ maturational timing were apparent [30], tight clustering of APHV values about the normative range (11.89 ± 0.54) was observed relative to the general population (12.00±1.0) in our female sample. However, when further analysed according to two sub-groups (10–13 v 14–15 years), a shift toward advantages favouring earlier maturing swimmers was apparent in the younger age-groups (11.70 ± 0.49); while in contrast, advantages towards later maturing swimmers were evident in later age-groups (12.22 ± 0.46). Findings suggest potentially transient advantages associated with maturity timing may occur in female swimming. That said, and with reference to *Aim 2* results, progressed maturational status still was beneficial to performance (see Fig 2). Although as a curvilinear relationship, benefits reduced with increasing maturity status. Overall, the combination of an absence in maturity timing bias across the whole sample, but with ‘Early’ maturity timing advantages in younger age-groups (until potentially post-APHV), suggests the presence of a counter-balancing relationship with 100-m FC performance.

Related to *Aim 3*, when examining raw performance times of the re-distributed maturity categories, findings identified significant overrepresentations of the ‘Early’ and ‘Early-normative’ maturing swimmers with large effect sizes at 10/11 and 12 years-old. The benefit of ‘Early’ maturity timing in younger age-groups was further emphasised when examining compositions of ‘Top 50%’ and ‘25%’ performance times. For instance, an ‘Early’ maturing 12-year-old swimmer was 32 times more likely to be in the ‘Top 25%’ relative to a ‘Late’ maturing swimmer (Table 2). Nevertheless, by 13 years-old, the shift in maturity timing advantages began, with no significant differences between any maturity timing category in the ‘Top 50%’ and ‘25%’ of performance times. By 14–15 years, overrepresentations of ‘Late-normative’ and ‘Late’ maturing swimmers—with medium-large effect sizes–were apparent. These findings again contrast with males, where the relatively older [27] and earlier maturing were consistently overrepresented until later youth age-groups (15–16 years), again supporting the notion of a counter-balancing relationship in females.

The observable differences between the present female sample and male youth swimmers could likely align with the differential impact of growth and maturation on anthropometric and physiological development. For instance, males typically experience more significant gains in height, muscular strength, and lean muscle mass, which positively influence swim performance [3, 19, 20, 40]. By comparison, while females experience height and strength gain (although often of less magnitude), they also experience gains in fat mass, and potentially, more varied changes in body shape (e.g., hip growth, torso shape change and breast development), which can negatively affect swim performance [23, 24, 41]. Thus, ‘Early’ maturing females before APHV may experience initial anthropometric and physiological performance advantages, but 4–6 months post-PHV (and onward) may experience maturity-related changes which may negatively impact performance [42] via influences upon propulsion, drag, and/or swim biomechanics.

Based on the curvilinear maturity status—performance relationship (*Aim 2*), when Mat-CAPs were applied to the 10/11-12 age-groups (*Aim 3*) and maturity category distributions re-examined, maturity-based performance advantages were successfully removed. There were no significant OR distributions between categories and across all selection levels. At 13 years old, a significant deviation did appear in the Correctively Adjusted ‘Top 25%’, although the sample size across the Top 25% of assessments should be considered. Given the present sample, the 13 years age-group seems to represent the chronological time-point where the benefits of advanced maturity status relative to age-group peers are potentially negated. Thus, from this time-point onward, delayed maturity timing may be advantageous in a proportion of cases. Overall, while females had contrasting maturity timing trends to males, akin to prior corrective adjustment studies [27, 30, 43], Mat-CAPs still mitigated against maturational bias until approximately 1-year post-PHV.

Notwithstanding the more nuanced findings, the present study is not without limitations. Present findings reside on a cross-sectional dataset, and longitudinal data would provide valuable verification, despite logistical and resource challenges. For example, the observed maturation timing and status distributions within age-groups may not have reflected true sample variation across female swimming. In contrast to prior studies, for *Aim 3*, maturity timing categories were created based on sample distributions and not normative population values. These maturity timing category ranges may not apply to alternative samples. Finally, suppose Mat-CAPs application was considered for other swimming strokes and distances (or other sporting contexts). In such cases, the sex and event-specific estimates of the maturation-performance relationship should be recognised, necessitating the need for independent data.

## Conclusion

Transient maturation status-based participation and performance (dis-)advantages were identified in a large sample of Australian female youth 100-m FC swimmers. In contrast to males, maturation-associated swim performance advantages in females occurred until approximately 13 years old, warranting restricted Mat-CAPs application. When applied to relevant age-groups, Mat-CAPs was able to remove maturational status inequalities across performance selection levels. Mat-CAPs illustrate the potential to improve youth female participation experiences and swimmer evaluation in specific early age-groups.

## Practical applications

- Swimming organisations, coaches and practitioners need to recognise and understand how growth and maturation can have dynamic positive and negative influences on participation and performance over time, depending on maturational timing and status relative to age-grouped peers.
- Based on an Australian sample and data, Mat-CAPs application could be utilised in female swimming, up to potentially 13 years old to account for inter-individual maturity-based influences on performance.
- The application of Mat-CAPs could help improve the accuracy of swimmer evaluation and assist in identifying swimmers’ with better technical proficiency given their developmental stage.
- Females with advanced maturational status before APHV may experience anthropometric and physiological performance advantages. However, in contrast to males, from post-PHV (e.g., 6 months) onwards influences may potentially be negative upon performance, due to anthropometric diversity and their impact upon propulsion, drag, and/or swim biomechanics.

## Supporting information

S1 Table Data summarising raw sample maturity timing distributions, chi-square, and odds ratio analyses of 663 female swimmers (10–15 years) in comparison to the expected distributions in the normative female population. (DOCX)

S1 Fig Maturity timing distributions of raw and correctively adjusted swim times in 100-m FC swimming according to selection levels for 12 years old. (DOCX)
