# Supplementary material for: Maturation-based Corrective Adjustment Procedures (Mat-CAPs) in youth swimming: Evidence for restricted age-group application in females
Source: PLoS One. 2022 Oct 7;17(10):e0275797. doi: 10.1371/journal.pone.0275797 (PMC9543692; doi:10.1371/journal.pone.0275797)
Supplement: S1 Table — (DOCX) [file pone.0275797.s001.docx]

**S1 Table.**

| **Raw population distribution** | **Age-Group** | **Total** | **Late** | **Late-Norm.** | **Early-Norm.** | **Early** | ***χ^2^*** | ***P*** | ***V*** | **ES cat.** | **Early v Late**  **OR (LCI-HCI)** | **Early-Norm. v Late**  **OR (LCI-HCI)** | **Late-Norm. v Late**  **OR (LCI-HCI)** |
| --- | --- | --- | --- | --- | --- | --- | --- | --- | --- | --- | --- | --- | --- |
| All swimmers | 10/11 Years | 124 | 2 | 25 | 90 | 7 | **85.62** | **<0.0001** | **0.48** | **Large** | 3.50 (0.64-18.99) | **20.91 (4.67-93.73)** | **5.81 (1.25-27.01)** |
|  | 12 Years | 153 | 1 | 45 | 100 | 7 | **80.20** | **<0.0001** | **0.42** | **Large** | 7.00 (0.8-61.28) | **46.48 (6.12-353.14)** | **20.91 (2.72-160.76)** |
|  | 13 Years | 147 | 3 | 71 | 72 | 1 | **58.01** | **<0.0001** | **0.36** | **Large** | 0.33 (0.03-3.44) | **11.15 (3.18-39.14)** | **11.00 (3.13-38.61)** |
|  | 14 Years | 144 | 4 | 89 | 50 | 1 | **69.59** | **<0.0001** | **0.40** | **Large** | 0.25 (0.03-2.41) | **5.81 (1.87-18.04)** | **10.34 (3.38-31.64)** |
|  | 15 Years | 95 | 6 | 76 | 13 | 0† | **91.42** | **<0.0001** | **0.57** | **Large** | 1.00 (0.02-53.76) | 12.08 (0.67-217.46) | **70.65 (4.10-1218.18)** |
|  | Total | 663 | 16 | 306 | 325 | 16 | **225.78** | **<0.0001** | **0.34** | **Large** | 1.00 (0.48-2.10) | **9.56 (5.51-16.63)** | **9.01 (5.18-15.67)** |

***Tables Notes:*** Late = Number with a late APHV (APHV >12.9 years); Late-Norm. = Number with a Late-Normative APHV (APHV < 12.9 years); Early-Norm. = Number with an Early-Normative APHV (APHV < 11.9 years); Early = Number with an Early Age of Peak Height Velocity (APHV < 10.9 years); † = Observed cell values of 0 were input as 0.5 to enable comparison of maturity timing categories; *χ^2^* = Chi-Square value; *P* = Probability value; *V* = Cramer's *V* effect size; ES cat. = Effect Size category; OR = Odds Ratio; LCI-HCI = Low & High 95% Confidence Intervals for maturation category comparisons; **bold** = Significant Chi-square (*p* < 0.05; with *P*, *V* and effect size category reported) and/or significant ORs (with LCI-HCI) in specific maturation status group comparisons.
