# Supplementary material for: Maturation-based Corrective Adjustment Procedures (Mat-CAPs) in youth swimming: Evidence for restricted age-group application in females
Source: PLoS One. 2022 Oct 7;17(10):e0275797. doi: 10.1371/journal.pone.0275797 (PMC9543692; doi:10.1371/journal.pone.0275797)
Supplement: S1 Fig — (DOCX) [file pone.0275797.s002.docx]

**S2 Fig.** Maturity timing distributions of Raw and Correctively Adjusted swim times in 100-m FC swimming according to selection levels for 12 years old.

***Figure notes:*** * = Significant deviation from reference category (Late) and relative to expected distributions (P < 0.05)
